# Space-like ^56^Fe irradiation manifests mild, early sex-specific behavioral and neuropathological changes in wildtype and Alzheimer’s-like transgenic mice

**DOI:** 10.1038/s41598-019-48615-1

**Published:** 2019-08-20

**Authors:** Bin Liu, Robert G. Hinshaw, Kevin X. Le, Mi-Ae Park, Shuyan Wang, Anthony P. Belanger, Shipra Dubey, Jeffrey L. Frost, Qiaoqiao Shi, Peter Holton, Lee Trojanczyk, Vladimir Reiser, Paul A. Jones, William Trigg, Marcelo F. Di Carli, Paul Lorello, Barbara J. Caldarone, Jacqueline P. Williams, M. Kerry O’Banion, Cynthia A. Lemere

**Affiliations:** 1 Department of Neurology, Ann Romney Center for Neurologic Diseases, Brigham and Women’s Hospital, Boston, MA 02115 USA; 2 Harvard Medical School, Boston, MA 02115 USA; 3 Harvard-MIT Division of Health Sciences and Technology, Massachusetts Institute of Technology, Cambridge, MA 02139 USA; 4 Department of Radiology, Brigham and Women’s Hospital, Boston, MA 02115 USA; 5 Department of Neuroscience, University of Rochester Medical Center, Rochester, NY 14642 USA; 6 GE Healthcare, Princeton, NJ 08540 USA; 7 GE Healthcare, Chalfont St Giles, HP8 4SP United Kingdom; 8 Harvard Medical School Mouse Behavior Core, Boston, MA 02115 USA; 9 Department of Environmental Medicine, University of Rochester Medical Center, Rochester, NY 14642 USA

**Keywords:** Inflammation, Behavioural methods, Alzheimer's disease, Astrobiology, Experimental models of disease

## Abstract

Space travel will expose people to high-energy, heavy particle radiation, and the cognitive deficits induced by this exposure are not well understood. To investigate the short-term effects of space radiation, we irradiated 4-month-old Alzheimer’s disease (AD)-like transgenic (Tg) mice and wildtype (WT) littermates with a single, whole-body dose of 10 or 50 cGy ^56^Fe ions (1 GeV/u) at Brookhaven National Laboratory. At ~1.5 months post irradiation, behavioural testing showed sex-, genotype-, and dose-dependent changes in locomotor activity, contextual fear conditioning, grip strength, and motor learning, mainly in Tg but not WT mice. There was little change in general health, depression, or anxiety. Two months post irradiation, microPET imaging of the stable binding of a translocator protein ligand suggested no radiation-specific change in neuroinflammation, although initial uptake was reduced in female mice independently of cerebral blood flow. Biochemical and immunohistochemical analyses revealed that radiation reduced cerebral amyloid-β levels and microglia activation in female Tg mice, modestly increased microhemorrhages in 50 cGy irradiated male WT mice, and did not affect synaptic marker levels compared to sham controls. Taken together, we show specific short-term changes in neuropathology and behaviour induced by ^56^Fe irradiation, possibly having implications for long-term space travel.

## Introduction

Humankind has not ventured beyond Earth’s protective magnetic field since Apollo 17’s twelve-day journey to the moon in 1972. Now, as NASA prepares for crewed voyages to Mars that would span years, space radiation still poses a substantial and poorly mitigated threat to humans in deep space. In NASA’s current design reference architecture for a Mars voyage, astronauts will receive approximately 1 Sievert of ionizing space radiation over the ~2.5 year mission^1,2^. This space radiation contains alpha particles, protons, and high charge and energy (HZE) particles, which are much more damaging to biological tissues than sparsely ionizing electromagnetic radiation such as gamma rays and X-rays. While spacecraft shielding can mitigate the dose received from solar protons and alpha particles, HZE particles either pass directly through the spacecraft or create showers of secondary radiation upon impact. Estimates suggest that each cell in an astronaut’s body would be traversed by some form of ionizing radiation every few days and specifically by an HZE particle every few months^3^. The highly damaging nature of even a single HZE particle traversal raises particular concern for tissues previously considered radioresistant, such as those of the central nervous system (CNS).

Studies in murine models have shown that exposure to high-energy ^56^Fe particles, which are the largest effective dose contributor in the space radiation environment^4^, impairs cognitive function. These impairments include deficits in spatial learning and memory, object recognition, and operant conditioning, which parallel the cognitive decline observed in aged animals^5–13^. For example, exposure to ^56^Fe particles at energies of 500 MeV per nucleon (MeV/u) or 1 GeV/u has been shown to impair hippocampal-dependent learning and memory performance on the Morris water maze^9–11,14^, the Barnes maze^5,7,8^, the 8-arm radial maze^15^, and contextual fear conditioning tests^6,12–14,16^. In fact, several investigations have reported cognitive impairment in animal models following doses of HZE radiation as low as 10–20 cGy^6,8,17,18^, which suggests that the doses relevant to NASA’s planned Mars mission are a significant health risk, and even lower doses are now being investigated^19,20^. These behavioural studies, taken together with investigations into low dose HZE radiation effects on neurons and neural tissue^21–23^, illustrate the risks that space radiation may pose to human health.

There has been surprisingly little investigation into the sex-dependence of radiation-induced pathology at the doses relevant to either clinical radiation therapies or crewed spaceflight missions, and it is important to note that the previously referenced work was performed predominantly in WT male animals. Nonetheless, radiation exposure limits suggested by the National Council on Radiation Protection are lower for females than for males, based on epidemiological studies of cancer incidence in humans^24^. However, these limits may not accurately reflect the risks of low-dose space radiation exposure. For example, the few studies investigating the sex-dependent neurobehavioural changes in mice after less than 1 Gy of X-rays or heavy ion exposure have shown less severe deficits in females than in males when compared to non-irradiated controls, which may be due in part to a neuroprotective effect of estrogen^25–27^. Notably, this particular sex-dependency seems to hold true only for low or fractionated doses. Studies using more than 1 Gy of gamma and ^56^Fe radiation indicate that female mice have a higher susceptibility to neurobehavioural changes in both WT and transgenic neurodegenerative disease models^12,28,29^.

In addition to sex differences, latent disease predilections may explain a significant portion of the variation in susceptibility to radiation-induced cognitive deficits, but research on the link between the two is in early stages. For example, the few studies that have investigated the interaction between radiation and the human alleles of apolipoprotein E (ApoE), a major genetic risk factor for AD, suggest that radiation exacerbates genotype-dependent neurobehavioural deficits in transgenic mice^28–32^. Like variations in ApoE, mutations in the amyloid precursor protein and presenilin are linked to AD, and HZE irradiation of mice with these mutations causes an acceleration of AD-related pathologies and behavioural deficits^6,33^. Furthermore, there are well-established connections between radiation exposure and oxidative stress^24^ and between oxidative stress and neurodegenerative disease^34,35^, suggesting that ionizing radiation could induce or exacerbate neurodegeneration in humans.

To date, the majority of these early studies have examined the effects of radiation in healthy, wild-type, male rodents. As the ultimate goal of this research is to mitigate space radiation damage to humans, it is imperative to thoroughly investigate how the effects of space radiation depend on major physiological variables such as sex and genetic predisposition to disease. Therefore, in the present study, we investigated the short-term, sex-specific effects of ^56^Fe radiation in a mouse model of AD amyloid and in wildtype littermates. Four-month-old male and female APP/PS1dE9 mice and WT littermates were exposed to 0, 10 or 50 cGy of ^56^Fe radiation and then analysed for neurobehavioural changes, chronic inflammation, and progression of AD-like pathologies. We report here that ^56^Fe particle irradiation results in sex-, genotype-, and dose-specific changes in hyperactivity, motor coordination, cognitive function, amyloid pathologies, and gliosis.

## Material and Methods

### Mice

**Figure 1:**
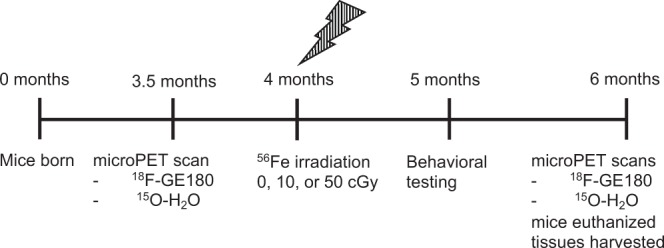
Study timeline. Mice underwent pre-irradiation ^18^F-GE180 and ^15^O-H_2_O microPET scans at 3.5 months of age before being transported to and from Brookhaven National Laboratory for irradiation at 4 months of age with 0, 10, or 50 cGy of ^56^Fe. Beginning at 5 months of age, mice underwent behavioral testing. At 6 months of age, mice underwent post irradiation ^18^F-GE180 and ^15^O-H_2_O microPET scans and were then sacrificed for tissue harvest and pathological and biochemical analyses.

This study used a total of 180 male and female APPswe/PS1dE9 Tg mice and age- and sex-matched C57BL/6J WT littermates. These Tg mice harbor the Swedish APP^K594N/M595L^ and PS1dE9 (deletion of exon 9) human transgenes under a mouse prion protein promoter^36^. The Tg mice have been shown to develop increasing levels of soluble Aβ oligomers, synaptic and neuritic pathology, Aβ plaque and vascular deposition, microhemorrhages, gliosis, and behavioural deficits with aging. These Tg mice develop extracellular amyloid deposits in cortex and hippocampus by 5–6 months of age^36,37^, and begin to exhibit cognitive deficits at 7–8 months^38^, which are exacerbated with age^39,40^. Mice were irradiated at 4-months of age at Brookhaven National Laboratory (BNL, Upton, NY). At the end of the study, the 168 remaining live mice were euthanized with CO_2_ at 6 months of age (Fig. 1). Mice were fed PicoLab^®^ Rodent Diet 20 5053 *ad libitum*. All experiments were conducted in accordance with the National Institutes of Health Guide for the Care and Use of Laboratory Animals and in compliance with all state and federal regulations. All animal use was approved by the Harvard Medical School Office for Research Subject Protection – Harvard Medical Area Standing Committee on Animals and the Brookhaven National Laboratory Institutional Animal Care and Use Committee.

### Irradiation of Mice with ^56^Fe

Tg mice and WT littermates were shipped to the animal facility at BNL in April 2015, allowed to acclimate for 3–5 days, and then transferred to the NRSL at BNL for irradiation. For the irradiation procedure, mice were individually loaded into 50 mL conical tubes with holes drilled for ventilation and then loaded into foam holders 10 at a time. The holders were carried into the exposure chamber and the mice were whole-body irradiated with 1 GeV/u ^56^Fe (actual energy 968.4 MeV/u, LET in water: 151.4 KeV/μm) at 10 or 50 cGy (13–16 mice per sex/genotype/dose) at a dose rate of 20 cGy/min (19.85–24.5 cGy/min). Particle fluence was 2,110,000 ions/cm^2^ for the 50 cGy exposures and 422,000 ions/cm^2^ for the 10 cGy exposures. Following a Poisson distribution, this equates to an estimated average 6.33 and 1.27 particle traversals through a 20 μm diameter circular target with unhit fractions (i.e. the probability of a target receiving no traversals) of 0.002 and 0.282 respectively. Upon completion of irradiation, mice were returned to their cages. Control mice were put into individual ventilated 50 mL conical tubes, loaded into the foam holders, and carried around the room for an equivalent period but were not taken into the exposure chamber.

### Neurobehavioural tests

A subset of 6–9 mice per group were analysed by a series of neurobehavioural tests (Table 1) between one and two months post irradiation (5–6 months of age). The SmithKline Beecham, Harwell, Imperial College, Royal London Hospital, phenotype assessment (SHIRPA) was used to measure baseline health and general function in mice as previously described^41^.

**Table 1:** Behavioural tests.

Behaviours	Tests
General Health	SHIRPA
Grip and Muscle Strength	Grip Strength (GS)
Depression	Tail Suspension Test (TST)
Anxiety	Open Field (OF) Elevated Plus Maze (EPM)
Locomotor Activity	Open Field (OF) Y Maze (YM) Elevated Plus Maze (EPM)
Motor Coordination	Rotarod
Motor Learning	Rotarod
Short-Term Memory	Y Maze (YM)
Fear Memory	Contextual Fear Conditioning (CFC)

#### Open field (OF)

measures changes in spontaneous locomotor activity, anxiety, and context habituation. Mice were placed into the centre of the test chamber (27 cm × 27 cm) and allowed to explore freely for 1 h. Locomotor activity was measured by a computer-assisted infrared tracking system that computes total distance traveled (cm) in 5 min time bins and counts for vertical exploration. The amount of time spent and distance traveled in the centre of the open field were also assessed as a measure of anxiety-like behaviour.

#### Y maze (YM)

spontaneous alternation test was used to assess spatial working memory performance by measuring the tendency of the mice to alternate arm entries, as well as to assess locomotor activity by measuring total arm entries and total distance traveled. Mice were placed into the centre of the Y maze and allowed to explore the environment for 6 minutes. The number and the sequence of arm entries were recorded.

#### Rotarod

was conducted to assess the mouse’s sensorimotor coordination and/or fatigue resistance. Following a habituation session (4 rpm, 5 min), Rotarod testing was performed at a steady rate of acceleration (4 to 40 rpm in 3 min). The latency to fall from the rod was recorded automatically. The test was repeated after 3 hours to assess motor learning.

#### Grip strength (GS)

was used to assess forepaw muscle strength by measuring the force required to pull the mouse off a narrow bar. The apparatus consisted of a grasping bar connected to a force transducer. The mouse was held by the base of the tail and placed in front of the grasping bar, which it would instinctively grab. The mouse was then slowly pulled back until the pulling force overcame the mouse’s grip strength. For each mouse, the test was run five times and the adjusted average grip strength was calculated after dropping the lowest and highest force measurements due to high variability intrinsic to the grip strength test.

#### Elevated plus maze (EPM)

consisted of two open and two closed arms extended out from a central platform. Each arm of the maze was 30 cm long and 5 cm wide. The maze surface was 90 cm above the floor, and the test was performed in dim ambient lighting. Mice were placed on the centre platform of the maze, facing an open arm, and were allowed to explore the apparatus for 5 minutes. A computer-assisted video-tracking system (TopScan software, CleverSys Inc., Reston, VA) was used to record the number of open and closed arm entries as well as the total time spent in open, closed, and centre compartments. An increase in the percent time spent in or entries into the open arms was used as a surrogate measure of anxiolytic behaviour. The number of closed arm entries was used as a measure of general locomotor activity.

#### Tail suspension test (TST)

was used to assess the anti-depressant efficacy of ^56^Fe irradiation in mice. Mice were suspended by their tails for 6 minutes and escape-related behaviours and immobility were assessed via automated Med Associates equipment. The MED Associates’ Tail Suspension Hardware uses a load cell to measure activity, and the load cell interfaces with the Tail Suspension Software via a PC to represent the activity as a voltage output. The time below threshold measure is an estimate of immobility time.

#### Contextual fear conditioning test (CFC)

was used to assess fear learning and memory and reflects the tendency of mice to freeze when re-exposed to the context where they received an aversive stimulus (footshock). Mice were allowed to explore the conditioning chamber (Med Associates) for 2 min and given two footshocks (0.5 mA; 2 s) separated by 2 min (training phase). Mice were removed from the chamber 1 min after the last foot shock. Twenty-four hours later, the mice were placed back into the conditioning chamber with no electric shock. The percent time spent freezing was video recorded over 3 min and scored by Topscan software (Cleversys). The freezing response was used as a surrogate marker of memory performance as mice that remember receiving the shock during the training phase on day 1 are expected to spend a significant amount of time freezing during the test phase on day 2.

### ^18^F-GE180 microPET Imaging for Neuroinflammation

Neuroinflammation status 2 weeks pre and 1.5 to 2 months post irradiation was monitored by PET imaging of translocator protein (TSPO) ligand radiolabeled with ^18^F-GE180. TSPO is an 18 kDa protein located in the mitochondria of glial cells that is maintained at a low level under normal conditions and is significantly upregulated during neuroinflammation.

#### ^18^F-GE180 radiotracer production

^18^F-GE180 was synthesized on the FASTlab^TM^ synthesizer using a previously published method^42^. Briefly, ^18^F was generated by proton irradiation of ^18^O enriched H_2_O (97% enrichment). After suitable workup the ^18^F anion was reacted with the precursor molecule (GE Healthcare), resulting in the formation of ^18^F-GE180.

#### *In vivo*^18^F-GE180 microPET imaging

In this study, four mice per group (0 and 50 cGy dose groups only) went through ^18^F-GE180 PET scans at 2 weeks prior to ^56^Fe irradiation and again from 1.5 to 2 months post irradiation to quantify changes in neuroinflammation. In each PET imaging session, mice were anesthetized with 3% isoflurane (Baxter Medica, AB) and medical grade oxygen at a rate of 1 L/min. After a CT scan, each mouse received the same dose per gram of body weight (1.75 µCi/g) of ^18^F-GE180 tracer solution by tail vein injection followed by a 0.1 mL saline flush. Dynamic PET imaging for each mouse brain was immediately performed for 60 min using a small animal PET/CT scanner (eXplore Vista, GE Healthcare). The spatial resolution of the PET scanner was 1.6 mm at the centre of the field-of-view (FOV). The data was acquired in 3D mode at the energy window of 250–700 keV, which yields 4% count sensitivity. The ^18^F-GE180 PET data was binned into 24 time frames (frames: 1 min × 8, 2 min × 6, and 10 min × 10) using Fourier rebinning (FORE) and reconstructed with ordered-subsets expectation-maximization (OSEM) algorithm with 16 subsets and 2 iterations. Random and scattered coincidence events were corrected during the image reconstruction. The voxel dimensions of the reconstructed images were 0.3875 × 0.3875 × ~0.775 mm^3^. The volume of interest (VOI) of whole brain or hippocampus was determined by co-registration of PET image, CT, and mouse atlas provided by VivoQuant imaging analysis program (Invicro, Boston, MA). The irradiation effects on whole brain and hippocampus-specific uptake (0–20 min) and stable binding (20–60 min) of ^18^F-GE180 PET tracer were quantified. Hippocampus-specific signal was normalized to thalamus-specific signal to control for inter-animal variability. Whole brain signal was not normalized due to lack of a low-signal reference tissue region in the scan, which included only head and upper thorax.

### PET Imaging for cerebral blood flow

#### ^15^O-H_2_O production

[^15^O]O_2_ was produced via ^14^N(d,n)^15^O reaction using a GE PETtrace 800 cyclotron running a 8 MeV deuteron beam at 40 µA on a ^14^N_2_/^16^O_2_ (1% O_2_) gas target. With the cyclotron operating in continuous run mode, the irradiated target gas was delivered to a GE ^15^O water process unit where it was combined with H_2_(g) and passed over platinum catalyst at 400 °C to form ^15^O-H_2_O. This vapor was bubbled into 8 mL of 0.9% saline in a vented 10 mL sterile vial until the desired activity was trapped (~8 min), giving 800–1000 mCi of ^15^O-H_2_O in 8 mL normal saline at the end of synthesis.

#### *In vivo*^15^O-H_2_O PET imaging

Tg and WT littermate mice (4 males and 4 females per genotype) underwent ^15^O-H_2_O microPET and CT imaging between 3.5 and 4 months of age, prior to ^56^Fe irradiation and again after irradiation at 6 months of age in order to quantify changes in cerebral blood flow (CBF). Each mouse was injected with 0.2 mL ^15^O-H_2_O water with 1.6 mCi of radioactivity. A 5 min dynamic half-body (including whole brain and heart) PET imaging followed by CT imaging was performed using a small animal PET/CT scanner (eXplore Vista, GE healthcare). The ^15^O-H_2_O PET image was reconstructed into 24 time frames for kinetic analysis. The volume of interest (VOI) was placed on the whole brain by co-registration with CT images using VivoQuant. For kinetic analysis of cerebral blood flow, one tissue compartment model (1-TCM) analysis was used to determine the CBF kinetic parameters. Heart ventricular blood flow (within VOI) was used as the reference region to determine CBF washin parameter K1 and washout parameter k2.

### Mouse euthanasia and tissue preparation

Mice were euthanized by CO_2_ inhalation and transcardially perfused with 20 mL PBS. The brains were then removed and divided sagittally. One hemibrain was fixed overnight in 4% paraformaldehyde (PFA) followed by cryoprotection with 10% and 30% sucrose solutions, and then embedded in Optimal Cutting Temperature (OCT) compound as previously described^6^. The other hemibrain was snap-frozen in liquid nitrogen and stored at −80 °C for biochemical analysis.

### MSD Multiplex Aβ triplex-38/40/42 protein Elisa

Snap-frozen whole hemispheres were homogenized in 5 volumes of Tissue Protein Extraction Reagent (T-PER) buffer containing a protease inhibitor cocktail (Roche, Indianapolis, Ind., USA). Homogenates were spun at 175,000 *g* for 60 min at 4 °C. The supernatant (T-PER-soluble fraction) was stored at −80 °C and the pellet was resuspended in 10 volumes of guanidine buffer (5 mol/L guanidine HCl, 50 mmol/L Tris, pH 8.0). Guanidine samples were mixed overnight at 4 °C and were centrifuged again at 175,000 *g* for 60 min at 4 °C. The supernatant (T-PER-insoluble fraction) was transferred, aliquoted and stored at −80 °C. Cerebral levels of Aβx-38, x-40, and x-42 were measured simultaneously using the Meso Scale Discovery (MSD) 96-well multi-spot Human/Rodent (4G8) Aβ Triplex Ultra-Sensitive Assay. Briefly, the well plate, patterned with capture antibodies against Aβx-38, x-40 and x-42, was blocked by 1% Blocker A solution at room temperature and shaken for 1 h then washed three times with 1x Tris Wash Buffer. Then, 25 μL/well of detection antibody (4G8) solution and 25 μL/well of either sample or calibrators were added together into the wells and incubated at room temperature for 2 h. The plate was washed three times with 1x Tris Wash Buffer and then 150 μL/well of 2x MSD Read Buffer T was added. The plate was read on an MSD Sector Imager immediately after the Read Buffer was added. MSD’s electrochemiluminescence detection uses a SULFO-TAG™ label that emits light upon electrochemical stimulation initiated on the electrode surfaces of multi-array and multi-spot microplates.

### Immunohistochemistry (IHC) and histology and quantification

As previously described^43^, 20 μm OCT-embedded frozen mouse brain sections were immunolabeled using the ABC ELITE method (Vector Laboratories, Burlingame, Calif., USA). Aβ pathologies were assessed by using a rabbit polyclonal anti-Aβ antibody, R1282 (1:1 K, a gift from Dr. Dennis Selkoe, Ann Romney Center for Neurologic Diseases, Brigham & Women’s Hospital). Fibrillar amyloid in plaques and blood vessels was visualised with 1% aqueous Thioflavin S (Thio S; Sigma-Aldrich). Gliosis was assessed with the following antibodies: anti-ionized calcium-binding adapter molecule 1 (Iba-1) rabbit polyclonal antibody (pAb) (a marker for all microglia/microphage, 1:500, Wako), anti-CD68 rat monoclonal antibody (mAb) (a phagocytic microglial/macrophage marker, 1:200, BD Biosciences), anti-TSPO rabbit mAb (a mitochondrial marker upregulated in neuroinflammation primarily in mouse microglia, 1:1000, Abcam), and anti- glial fibrillary acidic protein (GFAP) mouse mAb (a marker for intermediate filament proteins expressed largely in astrocytes, 1:1000; Sigma-Aldrich). Hemosiderin staining using 2% ferrocyanide (Sigma) in 2% hydrochloric acid was used to detect microhemorrhages. Quantification of R1282 immunoreactivity (IR), Thio S, and gliosis markers was performed using BioQuant image analysis (Nashville, Tenn., USA). The threshold of detection was held constant during analysis. The percent area occupied by R1282, Iba-1, TSPO, CD68 or GFAP labeling in the entire hippocampus (HC) and/or frontal cortex (FC) was calculated for 2 equidistant sagittal sections (300 μm apart) per mouse. Thio S labeling was averaged across 3 consecutive sections in the middle plane of the hemibrain. The number of microhemorrhages was counted and averaged over 6 sections (3 consecutive sections, 2 planes) from each mouse. For each IHC or histological analysis, n = 6–9 mice.

### Western blot analysis for synaptic markers

Western blot was performed as we reported previously^41^. Briefly, T-PER soluble brain homogenates were separated on 12% Bis-Tris gels (Invitrogen). Proteins on the gels were transferred to polyvinylidene fluoride (PVDF) membranes and probed with antibodies recognising presynaptic markers: synaptophysin (SYP, 1:3000, Sigma) or vesicular glutamate transporter 2 (VGluT2, 1:3000, Millipore), and postsynaptic markers: postsynaptic density protein 95 (PSD-95, 1:2000, Millipore) or Homer-1 (1:1000, RD Systems). Anti-GAPDH antibody (1:3000, Abcam) was used as a protein loading control. After blocking and incubation with primary antibodies overnight, IRDye-labeled secondary antibodies were used to visualise protein bands of interest. The intensity of protein bands was analysed by Odyssey imaging system (Li-Cor).

### Statistical analysis

All data are expressed as mean ± SEM. A value of p < 0.05 was considered significant and p < 0.1 was considered a notable trend for all statistical tests. Behaviour test data were analysed in StatView 5.0 using 3-way ANOVAs for sex, genotype, and dose followed by comparisons within sex/genotype groups (i.e. between 0, 10, and 50 cGy of one sex/genotype combination) and between same-sex nonirradiated controls using Fisher’s Protected Least Significant Difference (PLSD). Non-behaviour data was analysed in Prism 8.0 (GraphPad) following a similar scheme but with Tukey’s multiple comparisons test for comparisons within sex/genotype groups and Student’s t- or Mann-Whitney U tests for comparisons between nonirradiated control groups. Data that failed normality (Anderson-Darling, D’Agostino-Pearson omnibus, and Shapiro-Wilk tests) or homoscedasticity (Spearman’s test) assumptions were analysed with multiple, fewer-dimensional ANOVAs (2-way within sex followed by 1-way within sex/genotype groups if needed), and corrections for unequal standard deviations and non-normal distributions were applied as appropriate. Survival data was analysed by the log rank test.

## Results

To investigate the early effects of ^56^Fe irradiation, female and male WT and Tg mice received 0, 10 or 50 cGy of 1 GeV/u ^56^Fe irradiation at 4-months of age (n = 13–16/group, whole body) (Fig. 1). MicroPET imaging (4 mice/group, 0 and 50 cGy groups) for neuroinflammation (tracer: ^18^F-GE180) and cerebral blood flow (CBF, tracer: ^15^O-H_2_O) was performed before and after ^56^Fe irradiation. Behavioural tests for general health, grip and muscle strength, locomotor activity, motor coordination and learning, depression, anxiety, spatial memory and contextual fear memory were conducted starting at 1 month after ^56^Fe irradiation on a subset of 6–9 mice/group that did not undergo imaging (Table 1). All mice were euthanized at 6 months of age. Blood was collected and brain tissue was harvested for biochemical and pathological analyses.

### Irradiation with ^56^Fe had little to no effect on mouse body weight or survival rate

Mouse body weight was recorded prior to shipment to BNL for irradiation and again immediately prior to euthanasia (Supplementary Fig. 1). Generally, male mice showed higher body weights than female mice, and irradiation showed no effect on mouse body weight in any groups except for a small but significant increase in Tg females exposed to 10 cGy (p = 0.02). Irradiation had no statistically significant effects on male or female mouse survival (Supplementary Fig. 1). Irradiation with 50 cGy had a small but significant decremental effect on the overall health (SHIRPA, Supplementary Fig. 2) of female Tg mice compared to those exposed to 0 and 10 cGy (p = 0.03 and 0.01 respectively).

### Irradiation with ^56^Fe changed locomotor activity, motor learning, grip strength, and cognition

**Figure 2:**
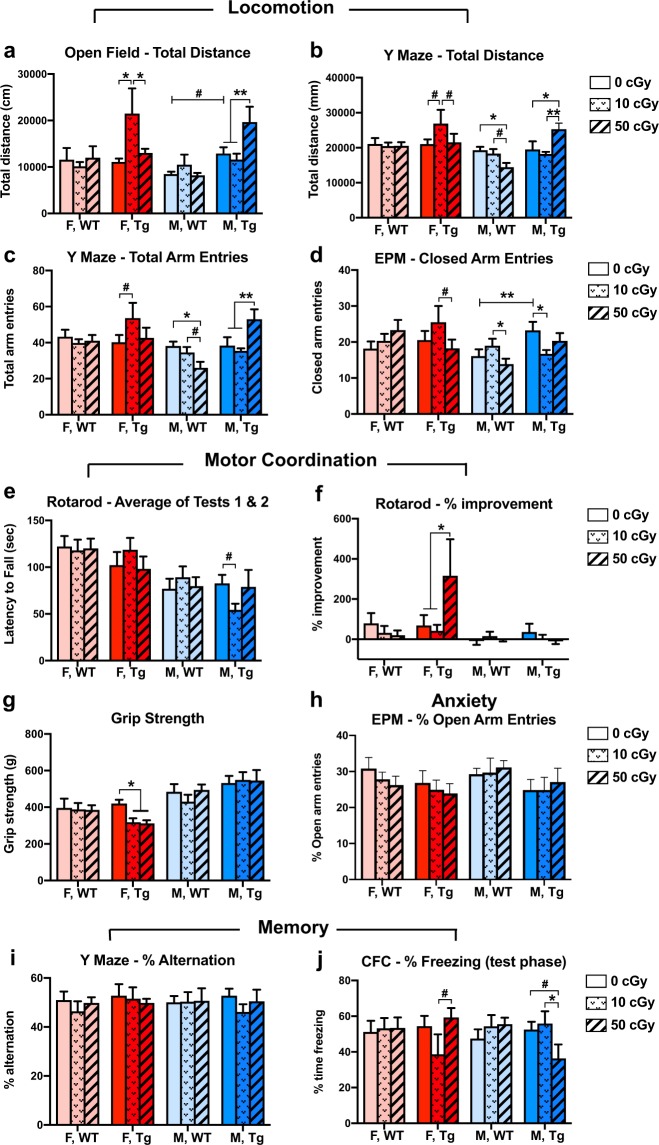
APPswe/PS1dE9 Tg and WT mice showed early neurobehavioral changes after a single dose of whole body ^56^Fe irradiation. Four-month-old female and male Tg mice and WT littermates were irradiated with 0, 10 or 50 cGy of ^56^Fe irradiation at BNL, and a subset of these mice were tested for behaviour at one month post irradiation. (**a–d)** ^56^Fe irradiation had sex- and genotype-specific effects on locomotor activity in open field-total distance (**a**), Y maze-total distance (**b**), Y maze-total arm entries (**c**), and elevated plus maze (EPM)-closed arm entries (**d**). (**e,f)** ^56^Fe irradiation did not affect motor coordination, except for a trend for a decrease in male, Tg, 10 cGy mice (**e**), but improved motor learning in female, Tg, 50 cGy mice (**f**). (**g)** ^56^Fe irradiation significantly and specifically lowered grip strength in female Tg mice. (**h–j**) ^56^Fe irradiation had no significant effects on anxiety (**h**) or spatial working memory (**i**), but it did produce sex-specific effects in the contextual fear conditioning (CFC) test in Tg but not in WT mice (**j**). F, WT: light red; F, Tg: dark red; M, WT: light blue; M, Tg: dark blue. n = 6–9 mice per group. Mean ± SEM; #p < 0.1; *p < 0.05; **p < 0.01.

The four locomotor activity behaviour tests (Fig. 2a–d) each showed significant 3-way interaction effects between sex, genotype, and dose (p = 0.004, 0.007, 0.005, and 0.016 respectively). As a whole, male mice appeared more susceptible to radiation-induced changes in locomotor activity than did female mice. In the open field test (Fig. 2a), nonirradiated Tg male mice showed a trend for higher locomotor activity than their WT counterparts (p = 0.06). Exposure to 50 cGy irradiation exacerbated this difference in the male Tg group, which was significantly more active than the 0 and 10 cGy groups (p = 0.008 and 0.002 respectively). Female Tg mice of the 10 cGy group showed a significant increase in activity compared to 0 and 50 cGy groups (p = 0.01, p = 0.04 respectively). No radiation-induced change in open field total distance was seen in male or female WT groups. Regarding locomotor activity in the Y Maze (Fig. 2b,c), 50 cGy irradiation reduced activity in male WT mice (total distance: 0 vs. 50 cGy p = 0.02, 10 vs. 50 cGy p = 0.06, trend; total arm entries: 0 vs. 50 cGy p = 0.01, 10 vs. 50 cGy p = 0.08, trend) but increased activity in male Tg mice (total distance: 0 vs. 50 cGy p = 0.01, 10 vs. 50 cGy p = 0.002; total arm entries: 0 vs. 50 cGy p = 0.007, 10 vs. 50 cGy p = 0.001). Female Tg mice showed a trend for increased activity in the 10 cGy group compared to 0 and 50 cGy groups in total distance (p = 0.07 and 0.09) and compared to 0 cGy in total arm entries (p = 0.06). Locomotor activity in the elevated plus maze (Fig. 2d) was elevated in nonirradiated Tg males compared to nonirradiated WT males (p = 0.008) as seen in the open field test. The dose-specific effects were more complex with Tg males showing a reduction in activity in the 10 compared to 0 cGy group (p = 0.02) but not in the 50 cGy group. WT males showed a reduction in activity in the 50 cGy group compared to the 10 cGy group (p = 0.04) but not compared to the 0 cGy group. Female Tg mice showed a trend for reduced activity in the 50 cGy group compared to the 10 cGy group (p = 0.09). These differences between 10 cGy and 50 cGy groups in the absence of differences from the nonirradiated controls may simply be a result of insufficient experimental power to discern the full relationship as irradiation often has been shown to have nonlinear effects on behaviour (e.g. elevated at lower doses but depressed at higher doses).

The rotarod tests showed a trend for impaired motor coordination after ^56^Fe irradiation (Fig. 2e) in 10 cGy male Tg mice compared to controls (p = 0.09) and significantly improved motor learning (Fig. 2f) in 50 cGy female Tg mice compared to 0 and 10 cGy groups (p = 0.03 and 0.02 respectively). ^56^Fe irradiation significantly reduced grip strength (Fig. 2g) in 10 and 50 cGy irradiated female Tg mice compared to nonirradiated controls (p = 0.03 and 0.02 respectively). No radiation effects were observed on anxiety-like behaviour (Fig. 2h). The tail suspension test (Supplementary Fig. 2), indicating depressive behaviour, showed a significant 3-way interaction between sex, genotype, and dose (p = 0.04), but no statistically significant comparisons between groups were seen. Male Tg nonirradiated mice showed a trend of less depressive behaviour (i.e. less immobility time spent below threshold) than WT counterparts (p = 0.06), and male Tg 50 cGy mice showed a trend of less depressive behavior than male Tg 10 cGy (p = 0.08). No radiation-induced change in depressive behavior was observed in any female or in any WT mice.

No radiation effects were observed on spatial memory in the Y maze (Fig. 2i). Interestingly, Tg mice but not WT mice were susceptible to radiation-induced changes in fear memory formation in the CFC test phase (Fig. 2j), which showed a trend for a 3-way interaction between genotype, sex, and dose (p = 0.06). Male Tg mice exposed to 50 cGy spent less time freezing than those exposed to 0 and 10 cGy (p = 0.07, trend, and 0.03, respectively). Female Tg mice exposed to 10 cGy spent less time freezing compared to those exposed to 50 cGy (p = 0.05), though the reduction did not reach threshold compared to the 0 cGy group due to high individual variability.

### Irradiation with ^56^Fe lowered ^18^F-GE180 uptake but did not alter neuroinflammation as assessed by microPET imaging

**Figure 3:**
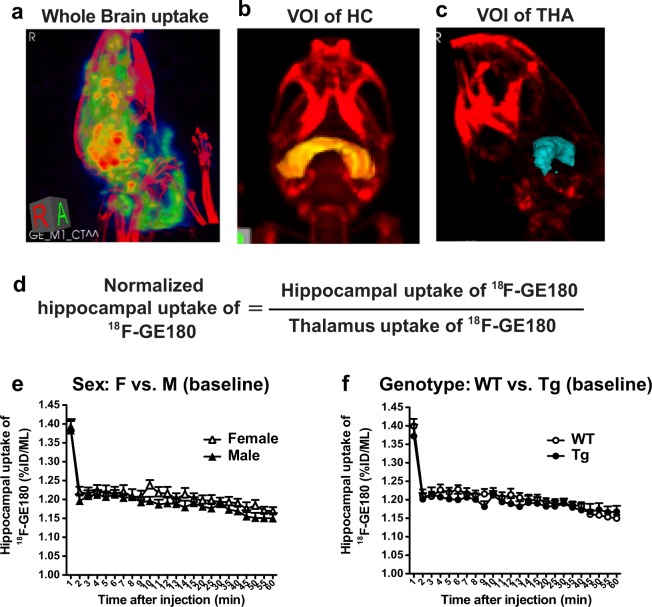
Brain inflammation was monitored by microPET imaging using a radiolabeled TSPO ligand, ^18^F-GE180. (**a–c)** To assess ^56^Fe irradiation effects on neuroinflammation, we analysed the tracer’s uptake and stable binding in whole brain (**a**), hippocampus (**b**) and thalamus (**c**). The thalamus was used as a reference brain region, due to lack of TSPO immunolabeling, as described in our previous study^44^. (**d**) The relative hippocampal uptake of ^18^F-GE180 was normalised by thalamus uptake. (**e,f**) No sex (**e**) or genotype (**f**) differences in hippocampal uptake of ^18^F-GE180 were found in pre-irradiation PET scans (baseline, n = 4 mice/group). Data analysed by 2-way ANOVA; mean ± SEM.

The early effects of radiation on brain inflammation and CBF were assessed by microPET using TSPO ligand tracer ^18^F-GE180 and ^15^O-H_2_O, respectively, 2 weeks before and 1.5 to 2 months after 0 or 50 cGy irradiation. Analysis of the pre-irradiation ^18^F-GE180 microPET scans indicated no sex or genotype differences in the baseline ^18^F-GE180 tracer uptake in whole brain (data not shown) or in hippocampus (Fig. 3e,f). The hippocampal uptake of ^18^F-GE180 was normalised to the radioactivity in the thalamus (Fig. 3d), which we had shown previously had minimal TSPO labeling by IHC^44^.

**Figure 4:**
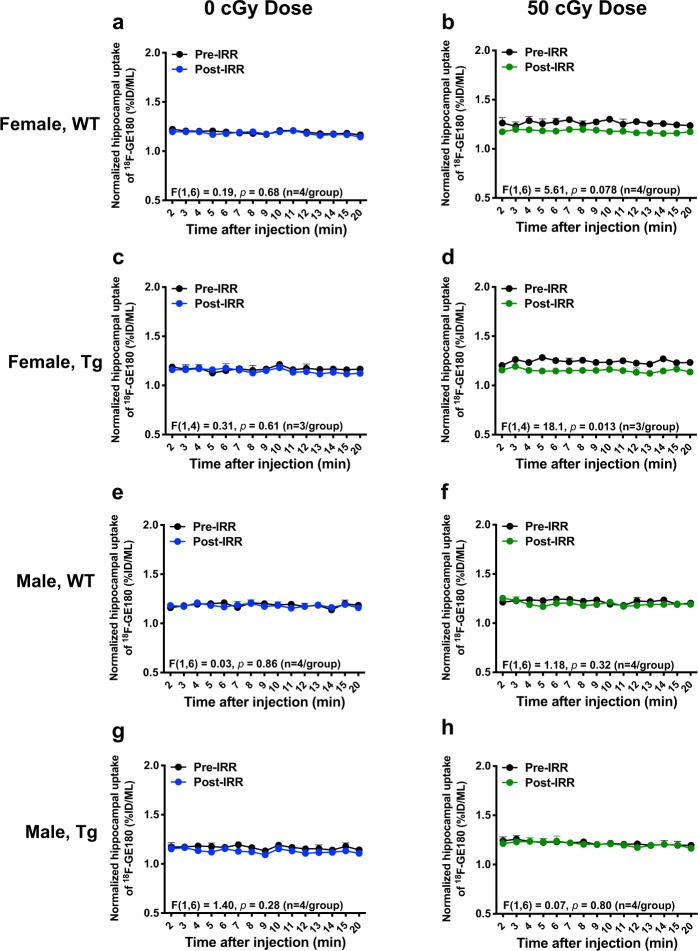
^56^Fe irradiation lowered hippocampal uptake of ^18^F-GE180 PET tracer in female but not in male mice. The uptake phase of ^18^F-GE180 was assessed from 0 to 20 min after tracer injection. (**a,c,e,g)** (left column) Pre-vs. post-irradiation PET signals of ^18^F-GE180 hippocampal uptake in 0 cGy irradiated groups. (**b,d,f,h)** (right column) Pre-vs. post-irradiation PET signals of ^18^F-GE180 hippocampal uptake in 50 cGy irradiated groups. Female WT and Tg mice irradiated with 50 cGy ^56^Fe had lower uptake of ^18^F-GE180 compared to baseline. n = 3–4 mice/group. Tracer level shown as percent injection dose per mL (%ID/ML). Data normalized to thalamus signal and analysed by 2-way ANOVA; mean ± SEM.

**Figure 5:**
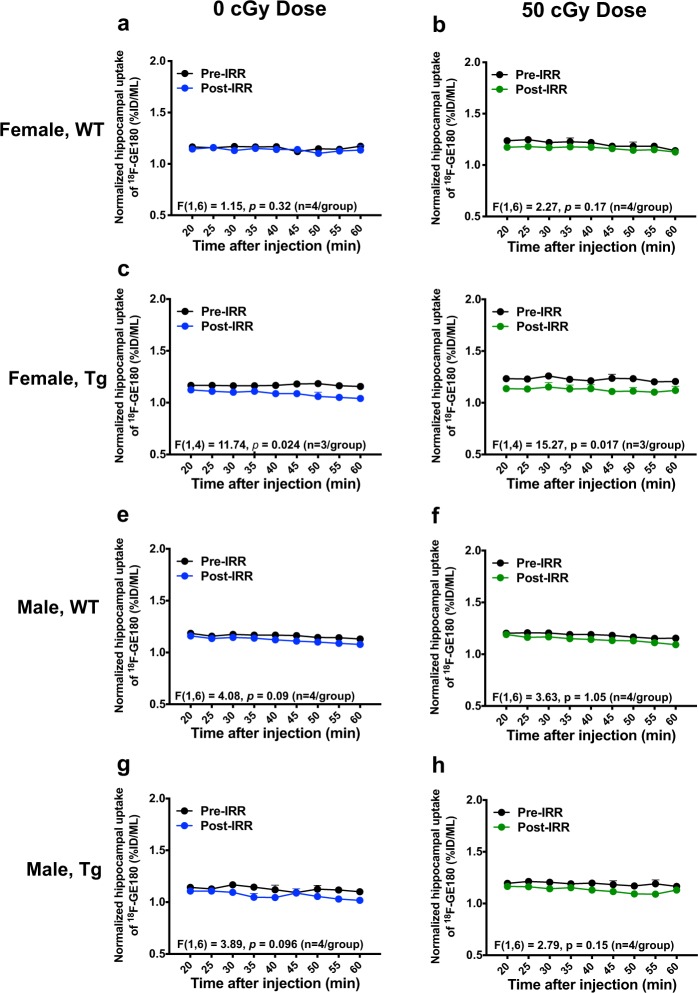
^56^Fe irradiation did not affect ^18^F-GE180 stable binding (neuroinflammation) in the hippocampus. The stable binding phase of ^18^F-GE180 was assessed from 20 to 60 min after tracer injection. (**a,c,e,g)** (left column) Pre-vs. post-irradiation PET signals of ^18^F-GE180 hippocampal stable binding in 0 cGy irradiated groups. (**b,d,f,h)** (right column) Pre-vs. post-irradiation PET signals of ^18^F-GE180 hippocampal stable binding in 50 cGy irradiated groups. Female Tg mice (0 and 50 cGy ^56^Fe groups) had lower uptake of ^18^F-GE180 compared to baseline but no radiation effects were seen. n = 3–4 mice/group. Tracer level shown as percent injection dose per mL (%ID/ML). Data normalized to thalamus signal analysed by 2-way ANOVA; mean ± SEM.

In agreement with our previous study^44^, uptake of ^18^F-GE180 in Tg and WT mice peaked ~8 min after tracer injection, while the tracer stable binding phase started ~20 minutes after tracer injection. Therefore, we divided the 60 min microPET scan into an uptake phase (0–20 min) and a stable binding phase (20–60 min), and performed further analyses for each phase. Irradiation with 50 cGy of ^56^Fe lowered hippocampal uptake of GE180 tracer in female WT mice (Fig. 4a,b, p = 0.078, trend) and in female Tg mice (Fig. 4c,d, p = 0.013) during the uptake phase, suggesting that ^56^Fe irradiation might inhibit the tracer’s uptake in a sex-specific manner. No effect was seen in any male mice (Fig. 4e–h). Interestingly, ^18^F-GE180 microPET analyses of the stable binding phase, which is associated with neuroinflammation, showed that the ^18^F-GE180 microPET signal at the later time point was slightly but significantly reduced in female Tg mice regardless of ^56^Fe irradiation (Fig. 5c,d). Stable binding phase analyses showed no radiation effect in female WT mice (Fig. 5a,b) or in any male mice (Fig. 5e–h) implying that ^56^Fe irradiation did not alter neuroinflammation 1.5 to 2 months after exposure. The results of hippocampal ^18^F-GE180 uptake and binding were corroborated by the whole brain ^18^F-GE180 uptake (Supplementary Fig. 3) and binding (Supplementary Fig. 4). CBF was assessed by ^15^O-H_2_O microPET scans pre and post ^56^Fe irradiation, and no significant radiation effects on CBF in any groups by 1-TCM analysis (Table 2) were observed, implying that ^56^Fe irradiation-related decreases in ^18^F-GE180 tracer uptake had no correlation with CBF after normalization to cardiac blood flow (Supplementary Table 1).

**Table 2:** Kinetic analysis of Vt, K1 and k2 for mouse cerebral blood flow pre and post ^56^Fe irradiation (IRR).

Sex	Genotype	Dose (cGy)	Vt (mL/cm^3^)		K1 (mL/cm^3^/min)		k2 (1/min)	
			Pre IRR	Post IRR	Pre IRR	Post IRR	Pre IRR	Post IRR
F	WT	0	0.70 ± 0.04	0.73 ± 0.03	3.10 ± 0.32	2.92 ± 0.35	4.20 ± 0.46	3.99 ± 0.44
		50	0.82 ± 0.05	0.78 ± 0.03	3.69 ± 0.64	2.95 ± 0.24	4.45 ± 0.63	3.80 ± 0.34
	Tg	0	0.75 ± 0.02	0.81 ± 0.03	2.17 ± 0.19	1.99 ± 0.47	2.91 ± 0.32	2.44 ± 0.53
		50	0.81 ± 0.11	0.77 ± 0.03	3.96 ± 1.37	2.31 ± 0.19	4.70 ± 1.30	3.02 ± 0.36
M	WT	0	0.71 ± 0.02	0.75 ± 0.03	2.88 ± 0.44	3.13 ± 0.64	4.07 ± 0.75	4.12 ± 0.73
		50	0.78 ± 0.03	0.75 ± 0.04	3.28 ± 0.89	3.49 ± 0.89	4.23 ± 1.15	4.58 ± 0.97
	Tg	0	0.73 ± 0.05	0.79 ± 0.07	4.03 ± 0.67	4.98 ± 2.05	5.60 ± 0.83	5.96 ± 2.07
		50	0.84 ± 0.02	1.10 ± 0.34	4.30 ± 1.37	3.91 ± 1.05	5.13 ± 1.57	4.60 ± 1.76

Mean ± SEM; no significant radiation-induced differences were observed.

### Irradiation with ^56^Fe reduced Aβ pathologies and gliosis in female Tg mice but not in male mice

**Figure 6:**
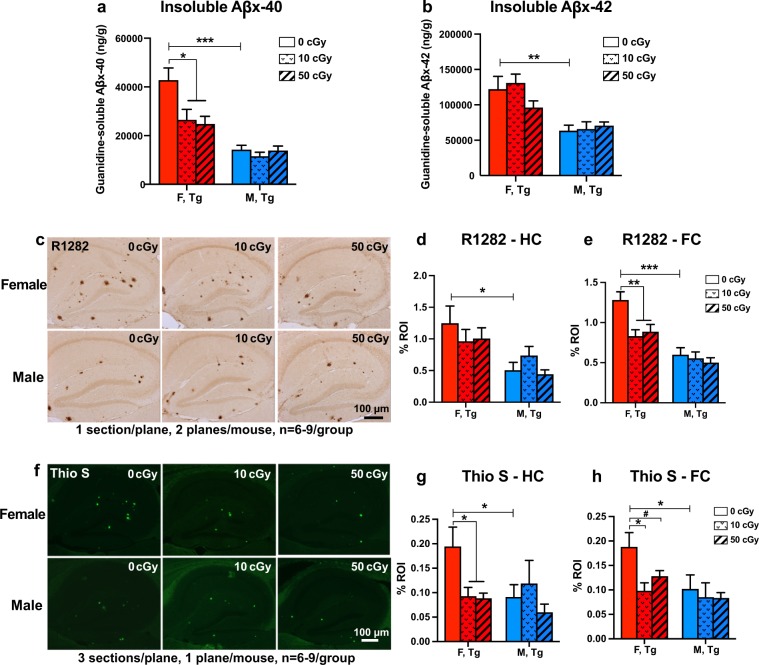
^56^Fe irradiation attenuated cerebral Aβ levels in female, but not male, APP/PS1dE9 Tg mice. (**a,b**) Insoluble Aβ x-40 (**a**) and x-42 (**b**) levels were quantified by MSD Aβ-triplex ELISA and are presented as nanograms per gram of total protein (n = 9–15 mice/group). Non-irradiated female Tg mice had higher Aβ levels than male Tg mice. Irradiation (10 and 50 cGy ^56^Fe) lowered insoluble Aβx-40 levels in female Tg mice only. (**c–e**) Amyloid deposition was assessed by R1282 immunohistochemical staining (**c**) and quantified by the % region of interest (%ROI) in the hippocampus (**d**) and frontal cortex (**e**) using BioQuant program (n = 6–9 mice/group). Nonirradiated female Tg mice had more Aβ deposition than male Tg mice. Irradiation with 10 and 50 cGy ^56^Fe reduced plaques in the frontal cortex of female, but not male, Tg mice. (**f–h)** Fibrillar Aβ was measured by Thioflavin S (Thio S) staining (**f**), and %ROI was quantified in the brain regions of hippocampus (**g**) and frontal cortex (**h**) using BioQuant (n = 6–9 mice/group). Fibrillar plaques were higher in female Tg than in male Tg mice. Irradiation with 10 and 50 cGy ^56^Fe lowered hippocampus and frontal cortex plaque load in female, but not male, Tg mice. Mean ± SEM; ^#^p < 0.1; *p < 0.05; **p < 0.01; ***p < 0.001.

The early effects of radiation on cerebral Aβ levels were assessed using a biochemical method (MSD ELISA, n = 9–15 per group) and quantitative immunolabeling with the anti-Aβ antibody R1282 and β-sheet structure dye Thio S (n = 6–9 per group). We observed that, without radiation, female mice in this Tg model developed significantly more Aβ pathologies earlier than males (Fig. 6), including higher: insoluble Aβx-40 (6a: p = 0.0002 by unpaired, two-tailed t-test with Welch’s correction), insoluble Aβx-42 (6b: p = 0.01 by unpaired, two-tailed t-test with Welch’s correction); Aβ immunoreactivity (R1282) in HC (6d: p = 0.02 by unpaired, two-tailed t-test) and FC (6e: p = 0.0002 by unpaired, two-tailed t-test); and, Thio S positive fibrillar amyloid in HC (6g: p = 0.04 by unpaired, two-tailed t-test) and FC (6h: p = 0.02 by Mann-Whitney U test). In female, but not male, Tg mice, a single exposure to ^56^Fe radiation lowered insoluble levels of Aβx-40 at both 10 and 50 cGy (Fig. 6a, p = 0.02 and 0.01 respectively), while no radiation-induced change was observed in insoluble levels of Aβx-42 in either sex (Fig. 6b).

Immunolabeling showed a similar pattern to the biochemistry data wherein radiation reduced pathology in female Tg mice but not in male Tg mice. R1282 immunolabeling showed a radiation-induced reduction in Aβ in the frontal cortex of females at both 10 and 50 cGy (Fig. 6e, p = 0.001 and 0.007 respectively) but not in the hippocampus (Fig. 6d). Thio S immunolabeling showed a radiation-induced reduction in β-sheet structure, which is associated with fibrillar Aβ plaques, in females at both 10 and 50 cGy in the hippocampus (Fig. 6g, both p = 0.02) and in the frontal cortex (Fig. 6h, p = 0.01 and 0.097, trend, respectively). This suggests that ^56^Fe irradiation specifically affected Aβ pathologies in female Tg mice but not in male Tg mice.

**Figure 7:**
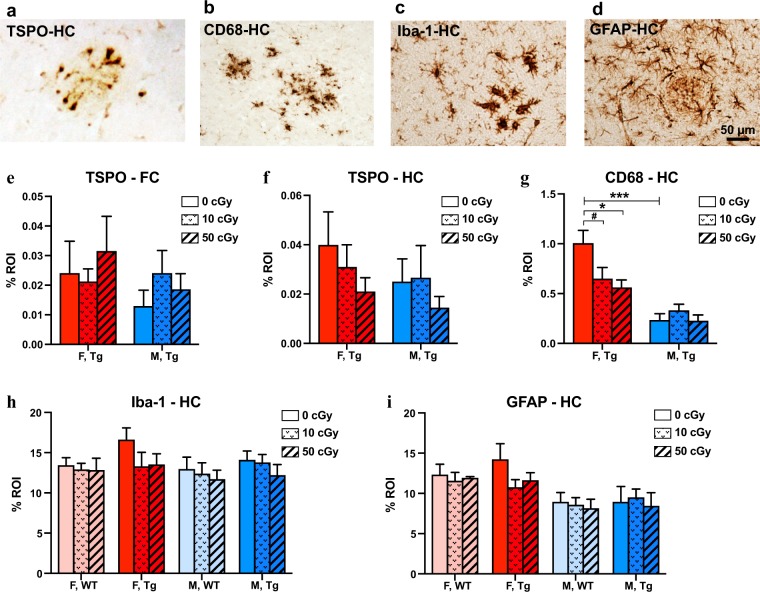
^56^Fe irradiation reduced CD68-associated gliosis in female, but not male, APP/PS1dE9 Tg mice. (**a–d)** Representative pictures of immunochemical staining for TSPO (**a**), CD68 (**b**), Iba-1 (**c**) and GFAP (**d**), respectively, on frozen brain sections of nonirradiated female Tg mice. (**e–i)** %ROI quantification of immunoreactivites of TSPO in frontal cortex (**e**) and hippocampus (**f**), as well as %ROI of hippocampal CD68 (**g**), hippocampal Iba-1 (**h**), and hippocampal GFAP (**i**). CD68-positive microglia/macrophage labeling was higher in nonirradiated female Tg mice than in male Tg mice. Irradiation with 10 and 50 cGy ^56^Fe reduced CD68 immuoreactivity in female, but not male, Tg mice. n = 6–9 mice/group. Mean ± SEM; ^#^p < 0.1; *p < 0.05; ***p < 0.001.

The lowering of Aβ in ^56^Fe-irradiated female Tg mice correlated well with reduced hippocampal microglial activation shown by CD68 staining (Fig. 7g). Following the same pattern seen in Aβ levels, nonirradiated Tg females had significantly higher CD68 staining than nonirradiated Tg males (p = 0.0001 by unpaired, two-tailed t-test), and 10 and 50 cGy irradiated Tg females had lower CD68 staining than nonirradiated Tg females (p = 0.07, trend, and 0.02 respectively). No radiation effects were seen in any groups by TSPO or Iba-1 microglia staining or by GFAP astrocyte staining (Fig. 7e,f,h,i). TSPO and CD68 immunoreactivity were too low to quantify in WT mice.

### Other ^56^Fe irradiation effects

**Figure 8:**
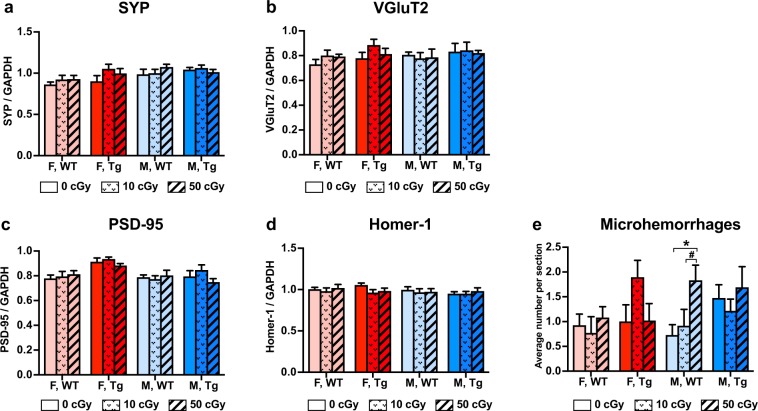
^56^Fe irradiation had no effect on whole brain levels of presynaptic and post synaptic markers but modestly increased microhemmorhages in male WT mice. Whole brain expression of pre-synaptic markers, synaptophysin (SYP) (**a**) and VGluT2 (**b**), and postsynaptic markers, PSD-95 (**c**) and Homer-1 (**d**), were measured by Western blot using mouse brain homogenates (n = 6–9 mice/group). No significant differences were observed between groups. (**e**) Microhemorrhages were visualised by hemosiderin staining on 6 brain sections per mouse and quantified by number. n = 6–9 mice/group. Mean ± SEM; ^#^p < 0.1; *p < 0.05.

Levels of pre-synaptic markers SYP and VGluT2 and of postsynaptic markers PSD-95 and Homer-1 were quantified by Western blot from whole brain homogenates (Fig. 8a–d). No early effects of ^56^Fe irradiation were observed on the whole brain levels of these synaptic markers. A small number of microhemorrhages were observed in all groups (e.g. 0–2 per section, Fig. 8e), but ^56^Fe irradiation increased the number of microhemorrhages in 50 cGy irradiated male WT mice compared to nonirradiated and 10 cGy irradiated WT males (p = 0.03 and 0.08, trend, respectively). ^56^Fe irradiation did not significantly affect the number of microhemorrhages in Tg mice or female WT mice.

## Discussion

Despite knowledge that spaceflight causes significant sex-dependent physiological changes in astronauts, the sex-dependent differences in neurobehavioural responses to HZE radiation are still relatively unexplored. This issue is not unique to the field of radiation biology. Including sex as a variable adds nontrivial work and complexity to study design, but its importance and benefits to biology at large are clear^45^. Additionally, data are limited regarding the interactions between radiation and genetic predispositions to disease. Most of the previous studies of ^56^Fe irradiation have used the same sex or same genotype of rodents (normally male WT) with high doses, conditions which are not representative of astronauts undergoing long-term space travel. Furthermore, recognizing generalized effects of low dose, heavy ion irradiation on behavior has been difficult, in part, due to variable individual susceptibility of mice and rats^8,46,47^. Recognizing these challenges, we nonetheless performed one of the first systematic investigations of short-term, sex- and genotype-dependent ^56^Fe irradiation effects on mice treated with doses relevant to spaceflight. Ultimately, we demonstrated that low dose ^56^Fe irradiation affected behavior in AD-like Tg mice more than WT mice and reduced AD-like early CNS pathologies in female, but not male, Tg mice.

We observed sex-, dose-, and genotype-specific ^56^Fe irradiation effects on locomotor activity, motor coordination, motor learning, and grip strength at ~1.5 months post ^56^Fe irradiation (Fig. 2a–g). In WT mice, 50 cGy irradiated males had significantly less locomotor activity compared to nonirradiated controls in the Y maze and compared to the 10 cGy exposure group in the EPM; no changes were observed in WT females. In Tg mice, radiation effects were seen in both males and females. Ten cGy irradiated Tg females and 50 cGy irradiated Tg males showed more locomotor activity compared to their respective nonirradiated control groups in the OF and Y maze tests. Notably, for female Tg mice, this radiation-induced increase in locomotor activity was not observed in the 50 cGy group. This low-dose-specific pattern was also observed in male Tg mice as a trend for reduced motor coordination. Furthermore, Tg mice showed sex-specific, radiation-dependent changes in motor coordination, motor learning, and grip strength while WT mice showed no changes in these abilities.

Previous behavioural studies using either WT mice or rats have shown little ^56^Fe irradiation effect on locomotor activities. Two-month-old female C57BL/6 mice irradiated with 0, 10, 50, or 200 cGy of ^56^Fe showed no behavioural changes in OF, rotarod, or acoustic startle at 2, 4, or 8 weeks post irradiation^48^. Male C57BL/6 mice exposed to 10 or 100 cGy of ^56^Fe irradiation showed no changes in locomotor activity or motor coordination at 5 and 9 months post irradiation^49^. ^56^Fe irradiation of male Sprague-Dawley rats at 150 cGy has been shown to cause anxiety-like behaviour in OF tests at 3 months post irradiation but did not affect locomotor activity^50^. Furthermore, 100–400 cGy ^56^Fe irradiation of 2-month-old male and female C57BL/6 mice showed no effects on locomotor activity 12 weeks post irradiation^12^. Note that most of the negative reports examined timepoints later than the 1–2 month time point investigated in our study. However, not all reports have shown no radiation effects. In a study of male and female B6F2D1 mice 3 months after irradiation with 25, 50, or 200 cGy of mixed ions (without ^56^Fe), male 50 cGy mice showed significantly higher activity levels in their home cages in the dark phase of their light-dark cycle^51^. Taken together, this suggests that a single behavioural test at a single time point may not be sufficient to uncover underlying radiation-induced changes, but nonetheless, mice with a neurodegenerative predisposition appear to be more susceptible than their WT counterparts to such changes.

Cognitive behavioural testing with the Y maze (spatial working memory) and CFC (contextual fear memory) showed no decline in WT mice at ~1.5 months after ^56^Fe irradiation (Fig. 2i,j). However, we did observe a trend of fear memory impairment in the ^56^Fe irradiated male, but not female, Tg mice even though these mice generally do not develop significant cognitive changes at 6 months of age^52–54^. Our findings were similar to those of a previous collaborative study with the O’Banion group, in which 3.5-month-old APPswe/PS1dE9 Tg mice were irradiated with 10 cGy or 100 cGy ^56^Fe, impaired fear memory was observed at later time points in males, but not females, following irradiation^6^. The lack of change observed in WT mice is consistent with some other investigations of HZE radiation effects^18,51,55^. However, many groups have consistently reported that low-dose HZE irradiation causes cognitive decline in spatial memory, fear memory, novel object recognition, and social function in WT rodents, especially at later timepoints^5,7,14,17,22,27,56–60^. These discrepancies, albeit among different rodent species and strains and with different testing protocols and environments, suggest that these deficits arise from a nuanced relationship with radiation type and/or post irradiation time point. It is also possible that, in the case of our study, percent alternation in the Y maze was not a sensitive enough test to capture early spatial memory deficits.

In the present study, no significant sex- or genotype-specific effects of ^56^Fe irradiation on anxiety-like or depression-like behaviour were found at these short-term time points (Fig. 2h, Supplementary Fig. 2), which is consistent with other studies^12,51^. Studies examining later time points show conflicting results regarding HZE-dependent changes in anxiety-like behaviour, which suggest a nuanced relationship to sex, dose, and particle type^27,28,49^. For example, one study reported an increase in anxiety-like behaviours in male but not female mice at 80 days post 15 or 50 cGy mixed ion irradiation, but no changes were observed in either sex at 45 days post irradiation^27^.

In our study, whole brain expression of pre-synaptic markers, SYP and VGluT2, and expression of postsynaptic markers, PSD-95 and Homer-1, were not altered at 2 months after a single exposure to ^56^Fe irradiation. This is counter to much of the literature, which often reports changes in dendritic structure and synaptic markers specifically in the hippocampus after HZE exposure^18,58,60^. It is possible that there were indeed radiation-induced changes in synapse structure underlying the altered behaviour in these mice but that assessing protein levels from whole brain homogenates was not sufficiently sensitive to detect the changes. It is not fully understood how space radiation impacts cognition and behavior, however a number of potential mechanisms may be involved including alterations in synaptic function and neurotransmitter release^61–63^, hippocampal neurogenesis^21,64,65^, ApoE allele^10,32,66^ and reactive oxygen species (ROS) overload^67,68^. In 1985, it was reported that brain-only irradiation with argon and iron particles at a dose of only 50 cGy caused morphological changes in synaptic density in the hippocampus of C57BL/6 mice^69^. Later, it was found that spatial learning and reference memory deficits induced by ^56^Fe irradiation were related to synaptic neurotransmitter release^15^. Supporting this, exposure to 60 cGy of 1 GeV/u ^56^Fe particles has been shown to cause persistent perturbation of glutamate release from rat hippocampal nerve termini and a reduction in the levels of the glutamatergic NMDA receptors NR1, NR2A and NR2B, which suggests that alterations in glutamatergic neurotransmission induced by low doses of ^56^Fe-particle radiation may lead to deficits in hippocampal-dependent learning and memory^62^. Exposure to ^56^Fe-particle radiation has also been shown by electrophysiology to inhibit normal synaptic plasticity in hippocampal CA1 neurons^33,70,71^. Moreover, there is evidence that ^56^Fe irradiation differentially modifies synaptic plasticity with respect to the presence of a peripheral immunological stressor, demonstrating that the irradiated CNS abnormally processes peripheral immune signals^72,73^. Taken together, these data suggest that 1 GeV/u ^56^Fe-particle radiation in the 20–60 cGy range may suppress synaptic plasticity and lead to cognitive impairment, which is supported by behaviour data from many of the same studies. In addition, ROS from ^56^Fe irradiation and the resulting mitochondrial dysfunction may impair hippocampus and frontal cortex. ROS can cause alterations in the neuronal environment and can eventually lead to hippocampal neuronal death, progressive neurodegeneration, and subsequent impairment of cognitive function^67^.

In the aforementioned study conducted by the O’Banion group, we reported that 100 cGy ^56^Fe irradiation elevated cerebral Aβ load in male APPswe/PS1dE9 Tg mice at 6 months post irradiation but did not affect Aβ in the female APPswe/PS1dE9 Tg mice at 4 months post irradiation^6^. However, in the present study, we show that lower doses (10 and 50 cGy) of ^56^Fe irradiation affected sex-specific changes in Aβ levels at only 2 months post irradiation (Fig. 6). Immunohistochemical analyses of Thio S and R1282, combined with biochemical assessment using MSD Aβ ELISAs, showed that 10 and 50 cGy of ^56^Fe irradiation reduced Aβ pathologies in the female Tg mice but not in the male Tg mice. Note that nonirradiated female Tg controls had higher baseline levels of Aβ than the nonirradiated male Tg controls. Taken together, our results show that the irradiation-induced changes in amyloid pathologies depend on sex and vary with dose and time post irradiation. Low doses of ^56^Fe irradiation may protect female Tg mice from the early development of Aβ pathologies while higher doses might exacerbate the pathology in males at later stages of the disease. As a notable aside, one study reported a reduction in number and size of Aβ plaques in males of a similar AD-like Tg mouse model at 2, 4, and 8 weeks after high-dose, head-only X-ray irradiation at 30 weeks of age^74^. This, however, is difficult to interpret alongside our findings because of the differences between head-only and whole-body irradiation and because X-rays and HZE radiation have qualitatively different biological effects.

The radiation response of microglia is of particular interest in the realm of radiation neurobiology because of the roles these brain-resident macrophages play in mediating and mitigating neural damage and maintaining homeostasis. In the current study, we found that single doses (10 or 50 cGy) of ^56^Fe irradiation had no early effects on glial pathology in male or female WT mice. However, in Tg mice, we did observe a radiation-induced reduction in microglia phagocytic activity by CD68 staining in females but not in males (Fig. 7g), which tracked with cerebral amyloid reduction. This agrees with our prior collaborative study with the O’Banion group that showed no radiation-induced change in CD68, Iba-1 or GFAP immunoreactivities in male Tg mice six months after exposure^6^. Other studies using different radiation doses have shown mixed results. Exposure of male C57BL/6J mice to a single, head-only dose of 50–400 cGy of 600 MeV/u ^56^Fe particles mildly increased the total number of activated and newly born microglia two months later^75^. In another study, exposure of male and female C57BL/6J × DBA2/JF1 mice to 25, 50, or 200 cGy of mixed ion radiation resulted in a significant increase in cortical CD68 two months after exposure only in 200 cGy irradiated females compared to nonirradiated controls^51^. Yet another study reported that, 100 days after 50 cGy of mixed ion irradiation, male but not female C57BL/6J mice had increased microglial activation by Iba-1 staining in the hippocampus^27^. These results depict a complex relationship between radiation type, sex, brain region, and disease state in the microglial response to radiation, and further investigation of this critical cellular response is needed in order to account for these diverse factors.

MicroPET imaging allows for the longitudinal study of mice from pre to post irradiation time points, which provides a more direct investigation of radiation effects than do cross-sectional designs. In this study, neuroinflammation was assessed by microPET imaging using TSPO-ligand PET tracer ^18^F-GE180. We found that ^56^Fe irradiation lowered ^18^F-GE180 uptake in female Tg and WT mice but did not affect the stable binding, a proxy for neuroinflammation, in any group. Although our IHC and PET imaging results were not directly comparable since the microPET results compared 0 and 50 cGy groups before and after irradiation while the IHC results compared 0, 10, and 50 cGy groups post irradiation only, they consistently indicated that ^56^Fe irradiation affected female Tg mice (decreased GE180 uptake, decreased gliosis, and decreased cerebral Aβ) more than the other experimental groups. The reason for the lowered cerebral ^18^F-GE180 PET tracer uptake in female Tg mice, particularly in high-dose irradiated female Tg mice, is still unclear, but it is probably not associated with cerebral blood flow (CBF), as ^15^O-H_2_O PET imaging indicated no radiation-induced change in CBF (Table 2). Despite this, the changes that occur in the early period after irradiation may be responsible for long-term effects seen months or years later. Although the incidence of microhemorrhage was low in these young mice, ^56^Fe irradiation slightly increased the number of microhemorrhages in male WT mice only. Further studies are underway using longitudinal MRI to look for radiation-induced microhemorrhages in WT and Tg mice exposed to a mixed field galactic cosmic ray simulation.

In conclusion, this study provides one of the first systematic evaluations of sex- and genotype-dependent early ^56^Fe irradiation effects on neurobehavioural changes, synaptic markers, amyloid pathologies, neuroinflammation, and cerebral blood flow in mice. We report significant effects of sex, genotype, and dose on changes in locomotor activity, motor coordination and learning, strength, contextual fear memory, AD-like pathology, and microglial activation within two months of irradiation. Many of these radiation-induced deficits were specific to Tg mice. We did not observe any radiation-induced changes in spatial memory, anxiety, neuroinflammation, cerebral blood flow, or whole-brain levels of synaptic markers in any groups. Importantly, female WT mice showed no radiation-induced changes while male WT mice showed only a mild reduction in locomotor activity and a modest increase in microhemorrhages, suggesting ^56^Fe had only mild early CNS effects in wildtype mice, which better represent healthy humans than do Alzheimer’s-like Tg mice. Additionally, we have investigated the sex- and genotype-dependent effects of ^56^Fe exposure at 8 months post irradiation, and those findings are currently being prepared as a separate paper. Altogether, the findings reported here add to the body of literature supporting the potential for mission-relevant doses of space radiation to impact CNS function. However, several caveats exist. For example, our studies do not reflect the chronic low dose radiation exposures (dose rate) expected on long term space missions where exposure to heavy ions become of even greater importance. In addition, familial AD mutations like those expressed in the mouse model used here are extremely rare whereas sporadic AD is much more common. In summary, though the risks to astronauts are not definitive, our work highlights the necessity of accounting for differences in sex and disease predilection in future research assessing and mitigating the risks of space radiation exposure.

## Supplementary information

Supplementary Materials

## Data Availability

The data generated during this study are available from the corresponding author on reasonable request.
