# Supplementary material for: Space-like 56Fe irradiation manifests mild, early sex-specific behavioral and neuropathological changes in wildtype and Alzheimer’s-like transgenic mice
Source: Sci Rep. 2019 Aug 20;9:12118. doi: 10.1038/s41598-019-48615-1 (PMC6702228; doi:10.1038/s41598-019-48615-1)
Supplement: Supplementary file 1 — Supplementary Materials [file 41598_2019_48615_MOESM1_ESM.pdf]

Space-like ^{56}Fe irradiation manifests mild, early sex-specific behavioral and neuropathological changes in wildtype and Alzheimer's-like transgenic mice

Author names

Bin Liu[1,2]*, Robert G. Hinshaw[1,2,3]*, Kevin X. Le[1], Mi-Ae Park[2,4], Shuyan Wang[4], Anthony P. Belanger[2,4], Shipra Dubey[2,4], Jeffrey L. Frost[1], Qiaoqiao Shi[1,2], Peter Holton[2,4], Lee Trojanczyk[5], Vladimir Reiser[6], Paul A. Jones[7], William Trigg[7], Marcelo F. Di Carli[2,4], Paul Lorello[8], Barbara J. Caldarone[8], Jacqueline P. Williams[9], M. Kerry O'Banion[5], Cynthia A. Lemere[1,2]

Affiliations

1. Department of Neurology, Ann Romney Center for Neurologic Diseases, Brigham and Women's Hospital, Boston, MA 02115, USA; 2. Harvard Medical School, Boston, MA 02115, USA; 3. Harvard-MIT Division of Health Sciences and Technology, Massachusetts Institute of Technology, Cambridge, MA 02139, USA; 4. Department of Radiology, Brigham and Women's Hospital, Boston, MA 02115, USA; 5. Department of Neuroscience, University of Rochester Medical Center, Rochester, NY 14642; 6. GE Healthcare, Princeton, NJ 08540, USA; 7. GE Healthcare, Chalfont St Giles, HP8 4SP, United Kingdom; 8. Harvard Medical School Mouse Behavior Core, Boston, MA 02115, USA. 9. Department of Environmental Medicine, University of Rochester Medical Center, Rochester, NY 14642.

*equal contribution

Corresponding author: Cynthia. A. Lemere, Ph.D., Ann Romney Center for Neurologic Diseases, Hale BTM Building, Room 9002S; 60 Fenwood Road, Boston, MA 02115; Tel: 617-525-5214; E-mail: clemere@bwh.harvard.edu.

Supplementary Table S1: Kinetic analysis of Vt, K1 and k2 for mouse heart blood flow pre and post ^{56}Fe irradiation (IRR)

Sex	Genotype	Dose (cGy)	Vt (mL/cm^3)		K1 (mL/cm^3/min)		k2 (1/min)	
			Pre IRR	Post IRR	Pre IRR	Post IRR	Pre IRR	Post IRR
F	WT	0	1.02±0.00	1.02±0.00	9.43±0.76	10.20±0.05	9.25±0.75	10.00±0.00
		50	0.95±0.06	1.02±0.00	8.41±1.68	10.18±0.02	8.67±1.33	10.00±0.00
	Tg	0	1.01±0.00	1.00±0.02	10.09±0.01	10.02±0.21	10.00±0.00	10.00±0.00
		50	1.03±0.01	1.02±0.00	6.15±0.06	10.19±0.02	6.00±0.00	10.00±0.00
M	WT	0	0.74±0.03	0.75±0.03	3.54±0.55	3.67±0.32	4.70±0.54	4.56±0.34
		50	0.77±0.03	0.76±0.03	3.82±0.92	3.88±0.98	4.89±1.13	4.99±1.17
	Tg	0	0.82±0.04	0.80±0.04	4.67±0.70	4.67±0.64	5.82±1.08	5.58±1.14
		50	0.80±0.02	0.81±0.02	2.86±0.60	3.26±0.65	3.54±0.64	3.74±0.92

Mean ± SEM

Supplementary Figure S1: Mouse body weight and survival rate

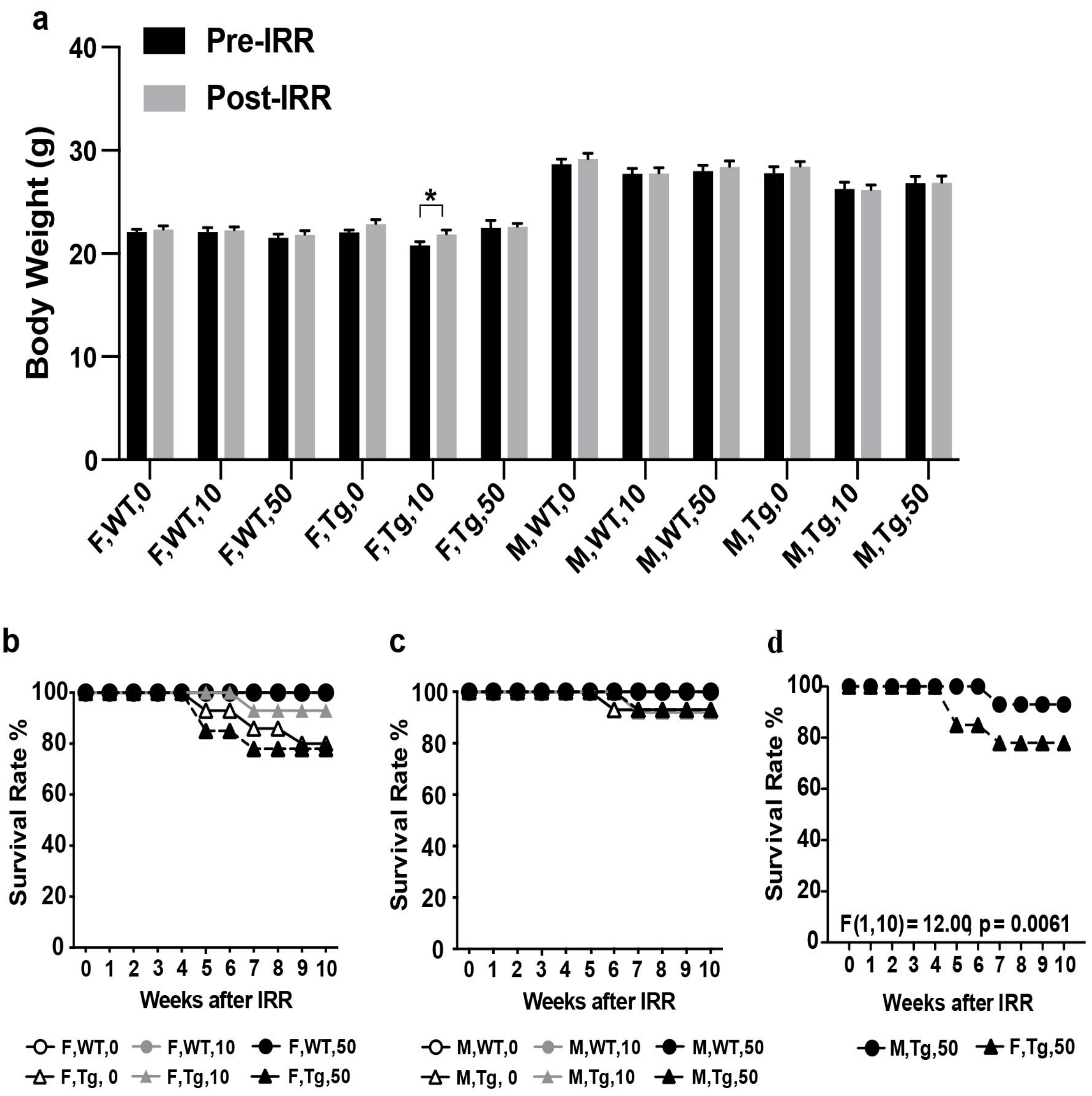

^{56}Fe irradiation had little to no effect on mouse body weight or survival rate. **a:** Mouse body weight was measured before shipment to BNL for irradiation (pre IRR), and again at 2 months after irradiation (post IRR). No change was observed except for a small but significant increase in the 10 cGy female Tg mice ($p=0.02$). Data were analysed by 2-way ANOVA; n=13-16 mice/group. **b-d,** ^{56}Fe irradiation had no significant effect on survival rate within male (**b**), female (**c**), or between male and female groups (**d**). The male Tg 50 cGy group lost one mouse over the course of the study, and the female Tg 50 cGy group lost three mice over the course of the study. Data were analysed by log rank test; n=13-16 mice/group. Mean ± SEM; $*p < 0.05$.

Supplementary Figure S2: Mouse general health and depression-like behaviour

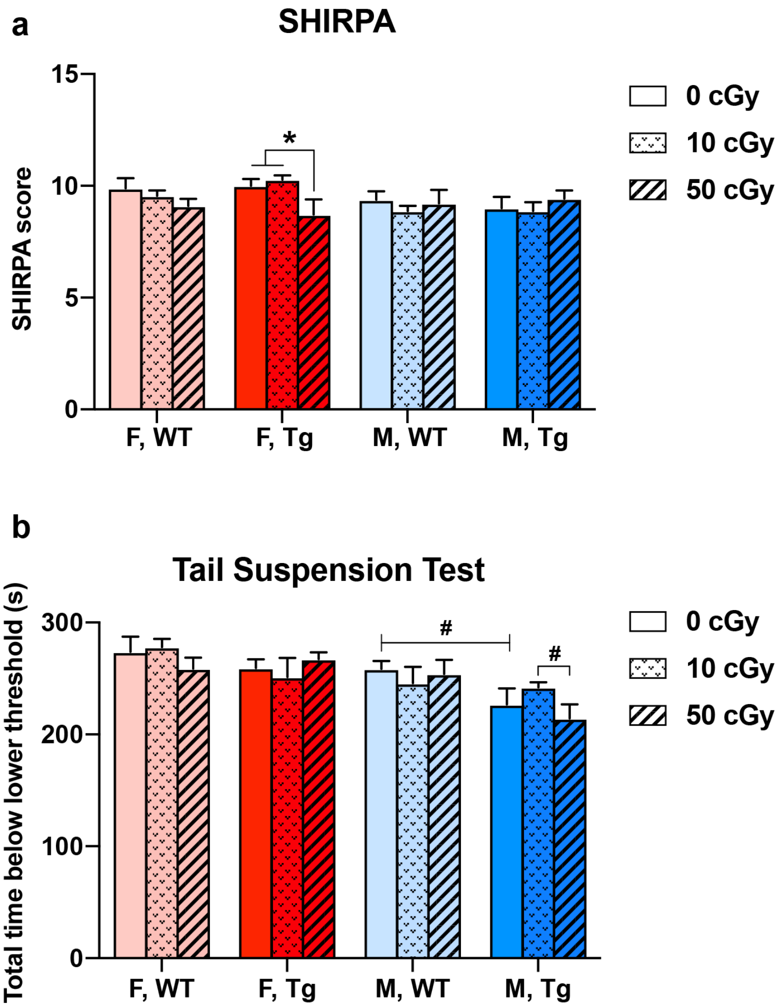

^{56}Fe irradiation had little effect on general health or depression-like behaviour (time below lower threshold). Four-month-old male and female Tg mice and WT littermates were irradiated with 0, 10 or 50 cGy of ^{56}Fe irradiation at BNL, and a subset of these mice (n=6-9 mice/group) went through behavioural tests starting at one month post irradiation. **a**: SHIRPA evaluation showed a small but significant radiation-induced decrement in general health in 50 cGy female Tg mice. **b:** Nonirradiated M Tg mice demonstrated less depression-like behavior than M WT mice. ^{56}Fe irradiation showed a modest trend for decreasing depression-like behaviour in the tail suspension test in 50 cGy compared to 10 cGy male Tg mice. Mean ± SEM; #p < 0.1; *p < 0.05.

Supplementary Figure S3: Whole brain uptake of ^{18}F-GE180 PET tracer

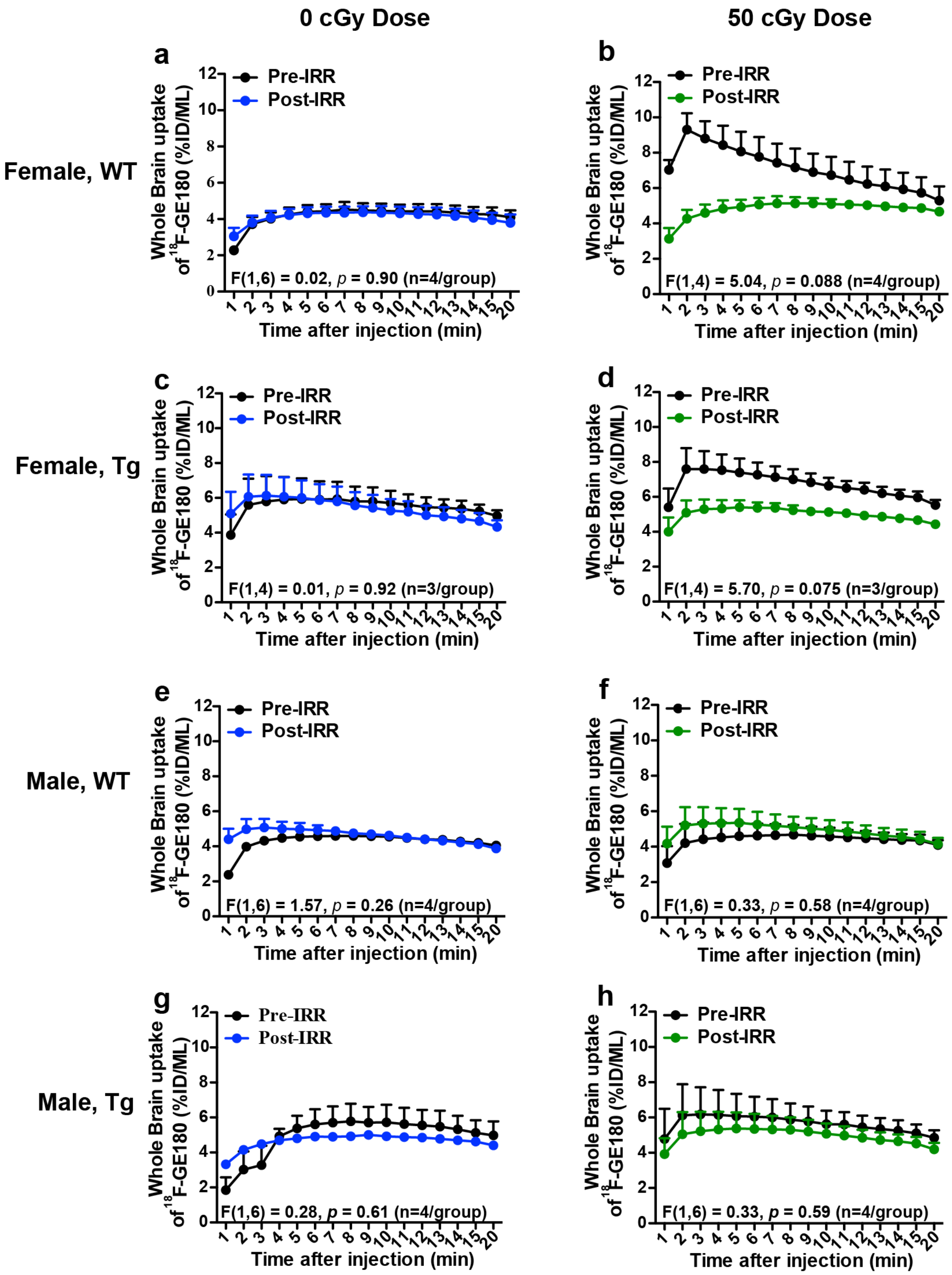

^{56}Fe irradiation lowered whole brain uptake of ^{18}F-GE180 PET tracer. The uptake phase of ^{18}F-GE180 was assessed from 0 to 20 min after tracer injection. **a, c, e, g (left column):** Pre-vs. post-irradiation PET signals of ^{18}F-GE180 whole brain uptake in 0 cGy irradiated groups. **b, d, f, h (right column):** Pre-vs. post-irradiation PET signals of ^{18}F-GE180 whole brain uptake in 50 cGy irradiated groups. Overall, no significant effects were seen however, female WT and Tg mice irradiated with 50 cGy ^{56}Fe showed trends for lower ^{18}F-GE180 uptake into whole brain compared to pre-irradiation baseline. n=3-4 mice/group. Note: Whole brain signal was not normalized by thalamus signal. Tracer level shown as percent injection dose per mL (%ID/ML). Data analysed by 2-way ANOVA; mean ± SEM.

Supplementary Figure S4: Whole brain stable binding of ^{18}F-GE180 tracer

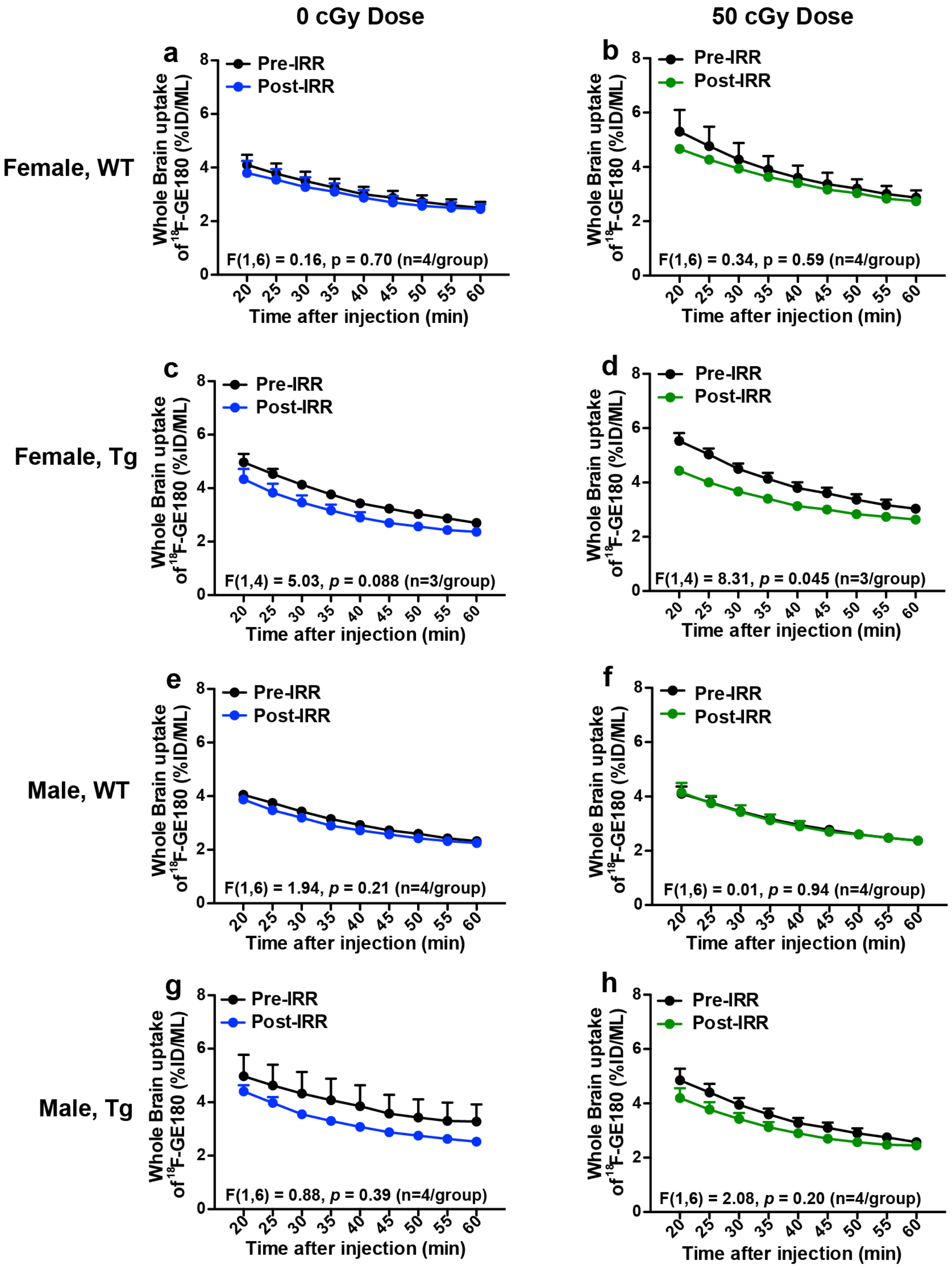

^{56}Fe irradiation did not affect ^{18}F-GE180 stable binding in the whole brain. The stable binding phase of ^{18}F-GE180 was assessed from 20 to 60 min after tracer injection. **a, c, e, g (left column):** Pre-vs. post-irradiation PET signals of ^{18}F-GE180 whole brain stable binding in 0 cGy irradiated groups. **b, d, f, h (right column):** Pre-vs. post-irradiation PET signals of ^{18}F-GE180 whole brain stable binding in 50 cGy irradiated groups. Post-irradiation whole brain stable binding of ^{18}F-GE180 was lower in female Tg mice (0 and 50 cGy) but only reached significance in the 50 cGy group. n=3-4 mice/group. Note: Whole brain signal was not normalized by thalamus signal. Tracer level shown as percent injection dose per mL (%ID/ML). Data analysed by 2-way ANOVA; mean ± SEM.
